# The R563Q mutation of the epithelial sodium channel beta-subunit is associated with hypertension

**DOI:** 10.5830/CVJA-2010-084

**Published:** 2011-10

**Authors:** Erika SW Jones, Brian L Rayner, E Patricia Owen, James S Davidson, Lize Van Der Merwe

**Affiliations:** Division of Hypertension, Groote Schuur Hospital and University of Cape Town, South Africa; Division of Hypertension, Groote Schuur Hospital and University of Cape Town, South Africa; Division of Chemical Pathology, Groote Schuur Hospital and University of Cape Town, South Africa; Division of Chemical Pathology, Groote Schuur Hospital and University of Cape Town, South Africa; LabPlus, Auckland Hospital, Auckland, New Zealand; Biostatistics Unit, MRC, Cape Town, South Africa

**Keywords:** hypertension, epithelial sodium channel, Liddle’s syndrome, R563Q mutation

## Abstract

**Background:**

A high prevalence of the R563Q mutation of the epithelial sodium channel β-subunit has been reported in South African hypertensives compared with unrelated normotensive controls. To delineate the effects of this mutation against a more uniform genetic background, this study investigated the association of the mutation with hypertension within affected kindreds.

**Methods:**

Forty-five index patients and members of their kindreds were studied. Blood pressure, serum potassium and the presence of the R563Q mutation were determined.

**Results:**

Of the 136 individuals studied, 89 were heterozygous for the R563Q mutation and 47 homozygous RR. The mean arterial pressure was significantly higher in the R563Q heterozygous group (*p* = 0.005) after adjusting for gender, race, age and kindred membership. Of the R563Q heterozygous subjects, 71 (80%) had hypertension, while 17 (36%) of the R563Q homozygous RR subjects were hypertensive. Six R563Q heterozygous subjects had hypokalaemia and one R563Q homozygous RR subject had hypokalaemia, but the difference was not statistically significant. Two heterozygous patients had Liddle’s syndrome, both occurring during pregnancy.

**Conclusion:**

The R563Q mutation of β-ENaC is associated with hypertension within affected kindreds, but does not usually cause the full Liddle’s syndrome phenotype.

## Summary

Increased activity of the epithelial sodium channel (ENaC) is the final common abnormality in several forms of hypertension: primary aldosteronism, glucocorticoid remediable aldosteronism, Liddle’s syndrome and 11-β-hydroxysteroid dehydrogenase-2 deficiency.^1^ Activating mutations of either the β- or γ-ENaC subunits can result in Liddle’s syndrome,^2^,^3^ in which constitutive reabsorption of sodium leads to hypertension and hypokalaemia in the presence of low plasma levels of renin and aldosterone.

We have previously reported the association of the R563Q mutation of β-ENaC with hypertension in black and mixed-ancestry South African patients, compared with unrelated normotensives.^4^ The purpose of the present study was to investigate the relationship within affected kindreds, who provided a more uniform genetic and environmental background against which the phenotypic effects of the mutation could be assessed.

The R563Q mutation is found in the intracytoplasmic terminal of the β-EnaC.^4^ This single nucleotide polymorphism substitutes glutamine for arginine at the 563rd amino acid. The variant allele under investigation in this study was glutamine and it is thought that the presence of glutamine in this position predisposes subjects to hypertension. The R563Q mutation was initially discovered by sequencing the carboxy terminal of the gene, and no other variant was found with the mutation in this region.

## Methods

The study was approved by the Research Ethics Committee of the University of Cape Town and all subjects gave signed informed consent. Index cases attending the hypertension clinic at Groote Schuur Hospital, and a normotensive subject with the mutation from the previous study^4^ and their kindreds were approached to participate in the study.

Blood pressure (BP) was measured in the sitting position, with a mercury sphygmomanometer, after five minutes’ rest. The mean of two stable readings was used. Hypertension was defined as systolic BP ≥ 140 mmHg and/or diastolic BP ≥ 90 mmHg, or the use of antihypertensive medication. The mean arterial pressure (MAP) was calculated for each patient as one-third of the pulse pressure plus diastolic pressure. Blood was then drawn for potassium (K^+^) and DNA analysis. Antihypertensive medication was not withdrawn prior to BP measurement or blood sampling.

DNA was extracted from whole blood. The carboxy terminal of the β-ENaC was amplified by PCR and the presence of the R563Q mutation was determined by Sfc-1 restriction enzyme digestion, and then confirmed by sequencing, as previously described.^4^

The distributions of some of the numerical variables were skewed, mainly due to extremes in the R563Q heterozygous group, so they were summarised as medians with interquartile ranges. Quantile normalisation^5^ was used to transform all to a normal distribution before analysis.

QTDT^6^,^7^ (http://www.sph.umich.edu/csg/abecasis/QTDT/index.html) was used to test for association between transformed numerical variables and R563Q mutation status. QTDT enabled us to take *degree* of relatedness into account in our analysis and hence adjust for the facts that firstly, offspring inherit their genes from their parents, with specific probabilities; and secondly, observations between family members are often more similar (correlated). Ignoring the similarity, relatives might yield spuriously significant results. QTDT incorporates variance components methodology to adjust for identity-by-descent probabilities between relatives. In order to eliminate the effect that differences in factors can have: gender, race and age between the R563Q groups, we adjusted our tests for them.

## Results

Overall 136 individuals (45 index cases and 91 family members) were studied, of whom 89 were heterozygous for the R563Q mutation and 47 homozygous RR. No subjects in this study were found to be homozygous for the variant allele. All patients and subjects were from either mixed ancestry (Cape Coloured) or Xhosa origins, and 67 (49%) were male. The demographics and other characteristics of the subjects are summarised in Table 1. The *p*-values in Table 1 are for a test of association with mutation status, after adjusting for gender, race, age and kindred membership. Effect estimates were not done because tests were performed on quantile-normalised variables, therefore only *p*-values are presented.

**Table 1.** Characteristics Of The R563Q Heterozygous And Homozygous RR Groups. Quantitative Variables Are Shown As Median And Interquartile (IQ) Range

	*R563Q heterozygous*						*R563Q normal*			
	*Index cases*			*Relatives*			*Relatives*			
	*Number*		*Percentage*	*Number*		*Percentage*	*Number*		*Percentage*	
Male	19/45		42	24/44		55	24/47		51	
Xhosa	14/45		31	8/44		18	7/47		15	
Mixed ancestry	31/45		69	36/44		82	40/47		85	
Hypertensive	44/45		98	27/44		61	17/47		36	
	*Number*	*Median*	*IQ range*	*Number*	*Median*	*IQ range*	*Number*	*Median*	*IQ range*	p*-value*
Age (years)	45	54	(24, 78)	44	39	(14, 39)	46	34	(26, 44)	
Systolic BP (mmHg)	45	159	(90, 270)	44	140	(92, 250)	47	132	(123, 149)	0.003
Diastolic BP (mmHg)	45	99	(60, 190)	44	82	(67, 155)	47	84	(73, 91)	0.01
MAP (mmHg)	45	121	(70, 217)	44	103	(77, 187)	47	101	(90, 110)	0.005
Serum potassium (mmol/l)	45	4.1	(2.1, 5.2)	39	4.2	(3.3, 4.9)	45	4.2	(4.0, 4.4)	0.224

*p*-values are for tests of association between the transformed variable and R563Q mutation status, after adjusting for covariates: gender, race, age and kindred membership, using QTDT. The number column for quantitative variables indicates the total number of individuals analysed for that variable.

Forty-four index cases had hypertension and one was a normotensive control identified in our previous study.^4^ Twenty-two index cases had at least one relative willing to participate in the study. Of the relatives, 72 were first-degree relatives (brother, sister, son, daughter, mother or father), 11 were nephew or niece, two were half brothers, two half sisters and four were grandchildren, in relation to the index cases.

There was a significant association between age and R563Q mutation status (*p* < 0.001), confirming the necessity of adjusting for age in all models. Heterozygous subjects were older than the homozygous RR subjects, with a median difference of nine years.

More R563Q heterozygous subjects, compared with homozygous RR, were receiving antihypertensive therapy, 66/89 (74%) versus 14/46 (30%) respectively. Of the R563Q heterozygous subjects, 71 (80%) had hypertension, while 17 (36%) of the R563Q homozygous RR subjects were hypertensive (*p* < 0.005). Only nine (14%) of the 71 heterozygous hypertensives were controlled on their antihypertensive therapy, versus two (12%) of the hypertensive homozygous RR subjects. Both mean systolic (*p* = 0.039) and MAP (*p* = 0.049) were significantly higher in the R563Q heterozygous subjects, despite antihypertensive treatment not being removed prior to examination.

Six R563Q heterozygous subjects had hypokalaemia. Two displayed the full Liddle’s syndrome phenotype during pregnancy, with severe unprovoked hypokalaemia (K^+^ = 2.1 and 2.2 mmol/l), marked suppression of aldosterone and renin, and hypertension. Three patients had hypertension and hypokalaemia associated with the use of diuretics, and another was normotensive with unprovoked hypokalaemia (K^+^ = 3.3 mmol/l). A R563Q homozygous RR family member had mild unprovoked hypokalaemia (3.4 mmol/l) and normal BP. However, the observed difference in the mean serum potassium between the R563Q heterozygous and homozygous RR groups was not significant (*p* = 0.224).

## Discussion

Historically, the genetic basis of hypertension has been a controversial issue. Platt (1947)^8^ proposed that essential hypertension is a Mendelian dominant trait with a distinct division between normotension and hypertension. On the other hand, Pickering (1955)^9^ proposed that hypertension was the extreme of the normal distribution of blood pressure and argued against having an arbitrary division between normotension and hypertension. He believed that hypertension has a multifactorial genetic and environmental basis with each individual trait having a small effect on blood pressure. The multifactorial nature of inheritance of blood pressure is now the accepted relationship between BP and genetics.

The β-ENaC, however, has always been an attractive candidate gene for a dominant form of hypertension since the discovery that Liddle’s syndrome was caused by an activating mutation in the β-subunit of the ENaC.^10^ However, the prevalence of Liddle’s syndrome was extremely rare, and, although several other more common mutations have been found, their causal relationship to essential hypertension is controversial.^11^-^23^

Our study examined the prevalence of hypertension and levels of blood pressure in families of R563Q heterozygous index cases and supports the theory that in the Xhosa and mixed-ancestry people in Cape Town, the R563Q mutation is an inherited cause of hypertension. A single copy of the variant allele of the R563Q mutation was significantly associated with hypertension: 71 (80%) of the R563Q heterozygous subjects had hypertension, while 17 (36%) of the R563Q homozygous RR subjects were hypertensive. Despite the small numbers of participants, families and individuals studied within the families, the association with hypertension was significant.

Further support for the causal nature of the R563Q mutation was demonstrated in the clinically significant differences in systolic, diastolic and mean arterial BP (Table 1). The differences in the medians for the heterozygous and homozygous RR groups were 18 mmHg, 8 mmHg and 12 mmHg for systolic BP, diastolic BP and MAP, respectively. Additionally, 74% of heterozygous patients were on antihypertensive treatment versus 30% for the homozygous RR group, which would tend to reduce the differences between the groups.

The R563Q mutation appears to be present in our population at a higher frequency than any of the other single nucleotide polymorphisms described to date which are causally linked to hypertension.^4^ In our specialist hypertension clinic, the prevalence of the mutation was 6.4% for black hypertensives and 4.6% for those of mixed ancestry, and currently all new patients are routinely tested for the mutation. The homozygous QQ genotype was not found in this study. This could be due to the small number of individuals studied, the small family sizes and the small number of families. The selection criteria for this study are also likely to have contributed to the lack of QQ homozygotes: heterozygotes known to the hypertension clinic were asked to be involved in the study.

The R563Q mutation is capable of causing the full Liddle’s phenotype, as was demonstrated in two patients who developed hypertension and severe persistent hypokalaemia during pregnancy. Only one relative was available for study in these kindreds and she was R563Q homozygous RR and normotensive. The presence of hypokalaemia in only a minority of the R563Q heterozygous subjects is consistent with the low penetrance of hypokalaemia reported in kindreds with other Liddle’s syndrome mutations,^24^,^25^ and suggests that other genetic or environmental factors are critical in determining the extent to which the full Liddle’s syndrome phenotype is expressed. Previously we reported this mutation in association with hypertension when it was found by sequencing the genes of the β- and γ-subunits of the ENaC in individuals with low renin hypertension.^4^ No other mutations were found when sequencing these subunits so it was deemed unnecessary to sequence these hypertensive patients.

There are no physiological data on the activity of the ENaC with this mutation. In Liddle’s syndrome, the R566X truncates the carboxy terminal of the β-EnaC, resulting in impaired internalisation of the channel and causing a persistently active sodium channel to reabsorb sodium. The R563Q mutation is three amino acids from the original Liddle’s mutation and may alter the protein from the positively charged arginine to the negatively charged glutamine and potentially alter the three-dimensional structure of the protein. Therefore, it is possible that this mutation decreases efficient intracellular interactions and internalisation of the EnaC, thereby increasing active channels on the apical membrane of the renal tubule. However, further physiological analyses are required to determine the differences in sodium channel activity due to the point mutation R563Q.

The R563Q mutation has not been detected in previous studies which included subjects of African descent.^26^,^27^ Further research into the geographical distribution of this mutation is required, particularly within the multi-ethnic black population of South Africa and Africa. The findings in our study are clinically relevant as it is anticipated that R563Q heterozygous individuals may respond to amiloride, a specific inhibitor of the EnaC.^28^

## Conclusion

We report the association of the R563Q β-ENaC mutation with hypertension within several kindreds. These findings strengthen the case for a causative role for R563Q in hypertension, because the genetic background is more uniform within kindreds than in the general population.
